# Adverse Perinatal Outcomes Among Teenage Mothers Delivering at a Tertiary Referral Hospital in Southwestern Uganda: Prevalence and Associated Factors

**DOI:** 10.7759/cureus.69040

**Published:** 2024-09-09

**Authors:** Joseph Ngonzi, Onesmus Byamukama, Wilson Birungi, Arnold Kamugisha, Moses Ntaro, Grace Nambozi, Leevan Tibaijuka, Lisa Bebell, Charles Tushabomwe-Kazooba, Kristien Roelens

**Affiliations:** 1 Obstetrics and Gynecology, Mbarara University of Science and Technology, Mbarara, UGA; 2 Community Health, Mbarara University of Science and Technology, Mbarara, UGA; 3 Nursing, Mbarara University of Science and Technology, Mbarara, UGA; 4 Medicine, Massachusetts General Hospital, Harvard Medical School, Boston, USA; 5 Central Administration, Mbarara University of Science and Technology, Mbarara, UGA; 6 Obstetrics and Gynecology, Women's Clinic, Ghent University Hospital, Ghent, BEL

**Keywords:** risk factors, stillbirth, neonatal, maternal-child, adverse perinatal outcomes, teenage pregnancy

## Abstract

Introduction: Each year, millions of teenagers in low-resource areas experience unintended pregnancies, many of which result in childbirth. These pregnancies often carry an increased risk of negative perinatal outcomes.

Objectives: The study determined the prevalence and factors associated with adverse perinatal outcomes among teenagers delivering at a tertiary referral hospital in southwestern Uganda.

Methods: This cross-sectional study was carried out in the Department of Obstetrics and Gynecology. We consecutively included all teenagers (13-19 years) in the postnatal ward who delivered. Descriptive statistics were used to summarize demographic and outcome data, and multivariable logistic regression analysis was used to identify factors associated with adverse perinatal outcomes.

Results: Overall, 327 participants were enrolled. The mean age was 18.4 (SD 1.1) years, while the mean number of antenatal care (ANC) visits attended was 4.6 (SD 1.9). Less than half delivered by cesarean 136 (41.6%) and 16 (4.9%) were HIV seropositive. Approximately 140 (42.8%) participants had adverse perinatal outcomes, including neonatal death (7, 2.1%), APGAR score at five minutes <7 (44, 13.5%), or low birth weight <2.5 kg (52, 15.9%). ANC attendance was mildly protective against adverse perinatal outcomes (aOR 0.91 (95% CI 1.14, 3.01), p=0.03). Feeling indifferent toward the pregnancy was associated with increased odds of one or more adverse perinatal outcomes compared to feeling happy about the pregnancy (aOR 3.39 (95% CI 1.11, 10.37), p=0.02). Participants with a history of prior miscarriage had increased odds of adverse perinatal outcomes (aOR 9.03 (95% CI 2.45, 25.53), p=0.04).

Conclusions: Nearly half of teenagers experienced adverse perinatal outcomes, and a history of prior miscarriage was a significant risk factor for adverse perinatal outcomes, while ANC was protective. Prospective cohort studies to explore the newborn and child developmental outcomes among children born to teenage mothers are also recommended.

## Introduction

Approximately, about 21 million teenagers aged 10-19 years in low-resource settings become pregnant every year. Approximately 50% of these pregnancies are unintended and result in an estimated 12 million births [1-2]. Teenage pregnancy is defined as pregnancy in those aged 10-19 years and is associated with many maternal and neonatal adverse effects such as premature deliveries, low birth weight, and pre-eclampsia [3-5]. Sub-Saharan Africa accounts for about half the global prevalence of teenage pregnancies and also approximately half of maternal, newborn, and child deaths [6-7].

Teenage pregnancy is a high-risk condition due to physiological and psychological immaturity, including insufficient sexual and reproductive knowledge, which leads to sub-optimal utilization of antenatal risk screening opportunities [8-9]. Teenage pregnancies are associated with major health and social challenges and can result in significant medical and psychosocial consequences for the adolescent and society [10-13]. Teenage pregnancies are specifically associated with a higher risk of preterm birth, preterm premature rupture of membranes, gestational hypertension, preeclampsia, low Apgar scores, anemia, intrauterine growth restriction, and stillbirths than non-teenage pregnancies [10,12,14-15].

There are multiple and complex contributing factors for teenage pregnancy, which are often categorized as socio-demographic, familial, cultural, and reproductive behaviors [16]. Factors associated with teenage pregnancy include living in rural areas, early marriage, low education level, lack of communication between parents and teenagers about sexual and reproductive health, and family history of teenage pregnancy [17-22].

However, there are gaps in knowledge around risk factors of teenage pregnancy, including the protective contribution of antenatal care (ANC), unsafe sexual practices with misperceptions, and misunderstandings about sexuality, conception, reproductive health, pregnancy, and contraception methods [23-24]. In order to help fill gaps in the literature, we aimed to establish factors associated with adverse perinatal outcomes among teenage mothers who delivered at Mbarara Regional Referral Hospital (MRRH) in Uganda.

## Materials and methods

Study design and setting

This cross-sectional study was carried out in the Department of Obstetrics and Gynecology at MRRH, a teaching hospital for Mbarara University of Science and Technology (MUST) with a catchment population of nine million people. The maternity ward performs about 9,200 deliveries annually, with a 40% cesarean delivery rate. Approximately 15% of women delivering at MRRH are referred from peripheral health facilities [25].

We included all teenagers (13-19 years) in the postnatal ward who delivered at MRRH. All pregnant women in the selected age range who presented to MRRH for delivery were consecutively approached for inclusion and enrolled after delivery. They were interviewed with standardized questionnaires.

Sample size calculation

The sample size was calculated using OpenEpi version 3 (www.OpenEpi.com). Using a proportion (28.6%) of teenage pregnancy taken from a study conducted in Ethiopia among female teenagers about prevalence and factors associated with teenage pregnancy to achieve a power of 80%, a margin of error of 5%, and a 95% confidence interval were assumed [26]. The sample size calculated was 327 participants.

Study procedures

After signing the informed consent form, a standardized questionnaire was administered to participants. Socio-demographic information, including age, gravidity, religion, marital status, number of ANC visits, employment status and level of education, and maternal and fetal outcomes, was obtained. Gestational age at delivery was calculated from the participant's reported last normal menstrual period (LNMP). When the participant was unsure of their LNMP, we relied on the first-trimester ultrasound scan in patients who came for ANC early in pregnancy. The following participant outcomes were abstracted from the participant charts. Pre-eclampsia/eclampsia, premature rupture of membranes, placenta previa, placenta abruption, perineal tear, and episiotomy. Adverse perinatal outcomes included preterm delivery, low birth weight (<2.5 kg), five-minute APGAR score <7, one or more birth defects, stillbirth, and neonatal death while the participant was still admitted to the maternity ward. A composite adverse perinatal outcome variable was defined as one of the adverse birth outcomes.

Ethical considerations

Ethical clearance was obtained from the Institutional Review Board of MUST (approval number: 09/05-17) and the Uganda National Council of Science and Technology (approval number: UNCST HS967ES). We also followed principles outlined in the Declaration of Helsinki on ethical principles for medical research involving human subjects.

Data management and analysis

Descriptive statistics were calculated as numbers, percentages, and frequencies. Categorical outcome variables were compared by exposure using Chi-square and Fisher’s exact tests. Multivariable backward stepwise analysis was performed using logistic regression, and only variables with a significance threshold of less than 0.2 were included in the final model. Measures of association were considered statistically significant at p<0.05.

## Results

The mean participant age was 18.4 (SD 1.1) years, while the mean ANC attendance was 4.6 (SD 1.9) visits, and the mean gestational age at delivery was 38.4 (SD 2.6) (Table 1).

**Table 1:** Mean and SD for participant age, ANC attendance, and gestational age SD: standard deviation, ANC: antenatal care

Participant variables	n (%)	
Age in years, mean (SD)		18.4 (1.1)
Number of ANC visits this pregnancy, mean (SD)		4.6 (1.9)
Gestational age at delivery, mean (SD)		38.4 (2.6)

Of 327 total participants, 224 (68.5%) were rural dwellers, 248 (75.8%) were married, 227 (69.4%) attained primary education, 200 (61.2%) had a family member who had been pregnant before the age of 20 years, 291 (89%) had not used contraception, 29 (8.9%) reported having had a miscarriage before this pregnancy, 24 (7.3%) reported alcohol use during the pregnancy, and 207 (63.3%) had a planned pregnancy. Overall, 16 (4.9%) were HIV seropositive, 171 (52.3%) had received prior sex guidance/counseling, and 136 (41.6%) delivered by cesarean (Table 2).

**Table 2:** Participant socio-demographic, medical, and obstetrical characteristics HIV: human Immunodeficiency virus

Participant variables		n (%)	
Religious affiliation			
Catholic		152 (46.5)	
Protestant		129 (39.4)	
Pentecostal		26 (8.0)	
Muslim		18 (5.5)	
Other		2 (0.6)	
Rural residence		224 (68.5)	
Marital status			
Single		70 (21.4)	
Separated/divorced		9 (2.8)	
Married		248 (75.8)	
Persons they live with (n, %)			
Husband		245 (74.9)	
Parent		43 (13.1)	
Relative/friend		33 (10.1)	
Alone		6 (1.8)	
Number of times pregnant, including the recently delivered one			
1		268 (82.0)	
2 or more		59 (18.0)	
Number of pregnancies carried beyond 7 months, including the recently delivered one			
0		4 (1.2)	
1		291 (89.0)	
2		32 (9.8)	
Employment status			
Not employed		251 (76.8)	
Employed		76 (23.2	
Level of education			
No education		11 (3.4)	
Primary education		227 (69.4)	
Secondary education		89 (27.2)	
No school attendance within the last 6 months		316 (96.6)	
Received sex guidance or counseling		171 (52.3)	
Another family member with pregnancy		200 (61.2)	
Prior contraception use		291 (89.0)	
No			
Current pregnancy planned		207 (63.3)	
Participant sentiment about this pregnancy			
Happy		212 (64.8)	
Unhappy		86 (26.3)	
Indifferent		29 (8.9)	
Self-reported HIV-positive serostatus		16 (4.9)	
History of prior miscarriage		29 (8.9)	
Alcohol use during the delivered pregnancy		24 (7.3)	
Delivery mode			
Normal vaginal		180 (55.0)	
Cesarean		136 (41.6)	
Assisted vaginal		11 (3.4)	

Most participants delivered at term (232, 70.9%), and 29 (8.3%) had a history of prior miscarriage. Overall, 140 (42.8%) experienced one or more adverse perinatal outcomes. Of 327 babies, 52 (15.9%) had low birth weight, 44 (13.5%) had a low APGAR score at five minutes, 11 (3.4%) were stillbirths, seven (2.1%) were in-hospital neonatal deaths, and 60 (18.3%) were delivered prematurely (Table 3).

**Table 3:** Adverse perinatal outcomes Kg: kilogram

Participant characteristics	n (%)
Gestational age at birth in weeks	
<37	60 (18.3%)
37-41	232 (70.9%)
≥42	35 (10.7%)
Birth weight <2.5kg	52 (15.9%)
Apgar score <7 at 5 minutes	44 (13.5%)
Birth defect	9 (2.8%)
Stillbirth	11 (3.4%)
In-hospital neonatal death	7 (2.1%)

The mean and SD for the participant age in the category of no adverse perinatal outcome was 18.5 (1.14) compared to 18.29 (0.96) in the participant category with adverse perinatal outcome. The mean and SD for the number of ANC visits in the category without adverse perinatal outcome was 4.73 (1.95) compared to 4.40 (1.90) in the category with adverse perinatal outcome (Table 4).

**Table 4:** Mean and SD for participant age and ANC attendance associated with one or more adverse perinatal outcomes SD: standard deviation, ANC: antenatal care

Participant variables	No adverse perinatal outcome (n=187)	Adverse perinatal outcome (n=140)	p-value
Age in years (mean, SD)	18.50 (1.14)	18.29 (0.96)	0.09
Number of ANC visits (mean, SD)	4.73 (1.95)	4.40 (1.90)	0.12

The composite adverse perinatal outcome was significantly associated with rural residence (p=0.03), living alone (p=0.001), primiparity (p<0.001), unemployment (p=0.03), unplanned pregnancy (p=0.002), and prior miscarriage (p<0.001, Table 5). Level of education, self-reported HIV serostatus, delivery mode, and current alcohol use were not associated with the composite outcome.

**Table 5:** Characteristics associated with one or more adverse perinatal outcomes HIV: human Immunodeficiency virus, ANC: antenatal care, SD: standard deviation

Participant characteristics	No adverse perinatal outcome, n (%)	Adverse perinatal outcome, n (%)	p-value
Residence type			
Rural	137 (73.3)	87 (62.1)	0.03
Urban	50 (26.7)	53 (37.9)	
Marital status			
Living alone	30 (16.0)	40 (28.6)	0.001
Separated/divorced	2 (1.1)	7 (5.0)	
Married	155 (82.9)	93 (66.4)	
Number of times pregnant			
1	166 (88.8)	102 (72.9)	<0.001
2 or more	21 (11.2)	38 (27.1)	
Employed			
No	152 (81.3)	99 (70.7)	0.03
Yes	35 (18.7)	41 (29.3)	
Level of education			
No education	7 (3.7)	4 (2.9)	0.45
Primary education	134 (71.7)	93 (66.4)	
Secondary education	46 (24.6)	43 (30.7)	
Prior contraception use			
No	168 (89.8)	123 (87.9)	0.57
Yes	19 (10.2)	17 (12.1)	
Planned pregnancy			
No	55 (29.4)	65 (46.4)	0.002
Yes	132 (70.6)	75 (53.6)	
Sentiment about the pregnancy			
Happy	134 (71.7)	78 (55.7)	<0.001
Indifferent	53 (28.3)	62 (44.3)	
HIV serostatus			
Negative	179 (95.7)	132 (94.3)	0.55
Positive	8 (4.3)	8 (5.7)	
History of prior miscarriage			
No	184 (98.4)	114 (81.4)	<0.001
Yes	3 (1.6)	26 (18.6)	
Current alcohol use			
No	173 (92.5)	130 (92.9)	0.91
Yes	14 (7.5)	10 (7.1)	
Mode of delivery			
Normal vaginal	106(56.7)	74(52.9)	0.788
Cesarean	75(40.1)	61 (43.6)	
Operative vaginal (e.g., vacuum extraction)	6(3.2)	5(3.6)	

ANC attendance was mildly protective against adverse perinatal outcomes (aOR 0.91 (95% CI 1.14, 3.01), p=0.03). Feeling indifferent toward the pregnancy was associated with increased odds of one or more adverse perinatal outcomes compared to feeling happy about the pregnancy (aOR 3.39 (95% CI 1.11, 10.37), p=0.02). Participants with a history of prior miscarriage were found to have increased odds of one or more adverse perinatal outcomes (aOR 9.03 (95% CI 2.45, 25.53), p=0.04). Delivery mode was not significantly associated with adverse outcomes despite the observed increased odds (Table 6).

**Table 6:** Logistic regression model of factors associated with an adverse perinatal outcome HIV: human immunodeficiency virus, OR: odds ratio, CI: confidence interval, ANC: antenatal care

	Univariable		Multivariable	
Characteristics	Crude OR (95% CI)	p-value	Adjusted OR (95% CI)	p-value
Age in years	0.83 (0.68, 1.03)	0.09	0.81 (0.72, 1.16)	0.30
Urban residence type	1.67 (1.04, 2.67)	0.03	1.23 (0.80, 2.01)	0.29
Pregnant 2 or more times	2.95 (1.64, 5.30)	<0.001	1.06 (0.48, 2.31)	0.82
ANC visits	0.91 (0.81, 1.03)	0.13	0.91 (1.14, 3.01)	0.03
Employed	1.80 (1.07, 3.02)	0.03	0.61 (0.93, 3.35)	0.15
Planned pregnancy	0.48 (0.30, 0.76)	0.002	0.57 (0.13, 1.46)	0.30
Feelings about the pregnancy				
Happy	1	1	1	
Indifferent	5.40 (2.21, 13.22)	<0.001	3.39 (1.11, 10.37)	0.02
HIV seropositive	1.36 (0.50, 3.71)	0.55	1.89 (0.73, 7.02)	0.20
History of prior miscarriage	14.0 (4.14, 47.27)	<0.001	9.03 (2.45, 25.53)	0.04
Alcohol use	0.95 (0.41, 2.21)	0.91	0.60 (0.30, 2.56)	0.62
Mode of delivery				
Vaginal	1	1	1	
Cesarean	1.17 (0.74, 1.83)	0.51	1.23 (2.12, 2.34)	0.34

## Discussion

In this study, we found that nearly half of adolescent pregnancies were associated with adverse perinatal events, including low birth weight, low APGAR score, stillbirth, neonatal death, and premature delivery. ANC attendance was somewhat protective against adverse perinatal outcomes, and feeling indifferent about the pregnancy was associated with increased odds of an adverse perinatal outcome compared to feeling happy about the pregnancy. A history of prior miscarriage was associated with adverse perinatal outcomes. Cesarean was also associated with increased odds of poor perinatal outcomes compared to normal vaginal delivery.

Similar rates of adverse birth outcomes have been reported among teenage mothers from many studies in similar settings [3,12,14-15,27-28]. Adverse birth outcomes among teenagers are thought to result from multiple factors, including physical immaturity, sub-optimal ANC, and possibly low partner involvement [1]. Poverty, low education, and inadequate family support may contribute to adverse perinatal outcomes such as preterm births and low birth weight [29].

In our study, ANC attendance was somewhat protective against adverse perinatal outcomes, a similar finding to several other studies [30-31]. ANC is the care provided by skilled healthcare providers to pregnant individuals to optimize health conditions for both mother and baby during pregnancy [32]. ANC helps in screening, diagnosis, and disease prevention among pregnant mothers, and by implementing timely and appropriate evidence-based practices, ANC can save lives [33]. Without optimal ANC attendance, the opportunities to diagnose diseases, including sexually transmitted infections that would cause adverse conditions such as premature labor, are missed.

In our study, cesarean delivery was associated with increased odds of adverse perinatal outcomes as compared to normal vaginal delivery. This is similar to what other studies have found [34-37]. It may be that cesarean delivery was performed for a specific maternal or fetal indication that impacted the well-being of the fetus and the newborn and resulted in an adverse outcome, including a low APGAR score [37]. Thus, cesarean delivery itself may be a marker of maternal or fetal disease or distress rather than a cause of adverse perinatal outcomes.

We also report an increased risk of adverse perinatal outcomes in a subsequent pregnancy for women with a history of prior miscarriage. Consistent with our findings, systematic reviews have previously reported an association between prior miscarriage and adverse perinatal outcomes [38-39]. This association is linked to the fact that a previous miscarriage predisposes women to placental dysfunction disorders, which in turn predisposes to adverse perinatal outcomes, including preterm labor and small for gestational age fetuses [40]. Previous studies [41] showed that miscarriage and placental dysfunction disorders have a shared pathogenesis of partial early placentation failure [42], resulting in a viable pregnancy but insufficient uterine spiral artery remodeling and a continued imbalance in angiogenic activity during the remaining pregnancy [43]. Another cause of adverse perinatal outcomes following a prior preterm birth may relate to premature cervical dilatation and abnormal initiation of labor [44-46].

Interestingly, in our population, feeling indifferent about the pregnancy was also associated with increased odds of an adverse outcome compared to feeling happy about the pregnancy. There is a paucity of data linking feelings toward pregnancy and adverse perinatal outcomes. However, teenagers who feel happy about the pregnancy may have had a planned pregnancy than those who feel indifferent or unhappy. Furthermore, those with unplanned pregnancies are twice as likely to have adverse outcomes such as low birth weight and preterm births as those with planned pregnancies [47-48], and there is an association between teenagers and unplanned pregnancies [49-50]. Those who are unhappy about the pregnancy may also be likely to adhere to the World Health Organization's recommendations about eight ANC visits, and most report for the first visit after 12 weeks [51]. Those with unplanned pregnancies may also start ANC late, resulting in inadequate ANC attendance, which may increase the risk of adverse perinatal outcomes [48,52-54].

Limitations of the study

During the interpretation of our research findings, the following limitations should be considered: The number and quality of ANC received were not explored, including the nutritional status of the teenage mothers, yet nutrition has a vital impact on outcomes. The height/weight, weight gain of the teenagers, the quality of ANC, Hb level, other prophylactic treatment adherence (iron, folate, calcium, vaccination), and diet were also not assessed in this present study.

The involvement of the partners and parents/guardians was not studied, such that the impact of their involvement was not known. In addition, although we report on alcohol consumption, we did not measure the use of other substances known to impact pregnancy outcomes, including tobacco. We also relied on the history of some important variables, such as alcohol use and HIV serostatus, rather than objectively testing the teenage mothers, which may have been subject to social desirability or recall bias.

Recommendations

Targeted prenatal and antenatal screening for causes of miscarriages in previous pregnancies is recommended. Prospective cohort studies to explore the newborn and child developmental outcomes among children born to teenage mothers are also recommended.

## Conclusions

Nearly half of adolescent pregnancies resulted in adverse perinatal events such as low birth weight, low APGAR score, stillbirth, neonatal death, or premature delivery. Cesarean delivery was associated with increased odds of adverse perinatal outcomes as compared to normal vaginal delivery. ANC attendance was found to be somewhat protective against adverse perinatal outcomes. Feeling indifferent about the pregnancy was associated with increased odds of adverse perinatal outcomes compared to feeling happy about the pregnancy.

## References

[1] Sully EA, Biddlecom A, Darroch JE (2023). Adding It Up: Investing in Sexual and Reproductive Health. Guttmacher Institute.

[2] Whitehead E (2009). Understanding the association between teenage pregnancy and inter-generational factors: a comparative and analytical study. Midwifery.

[3] Ganchimeg T, Ota E, Morisaki N (2014). Pregnancy and childbirth outcomes among adolescent mothers: a World Health Organization multicountry study. BJOG.

[4] Maheshwari MV, Khalid N, Patel PD, Alghareeb R, Hussain A (2022). Maternal and neonatal outcomes of adolescent pregnancy: a narrative review. Cureus.

[5] McCarthy FP, O'Brien U, Kenny LC (2014). The management of teenage pregnancy. BMJ.

[6] Kurth F, Bélard S, Mombo-Ngoma G (2010). Adolescence as risk factor for adverse pregnancy outcome in Central Africa--a cross-sectional study. PLoS One.

[7] Odimegwu C, Mkwananzi S (2016). Factors associated with teen pregnancy in sub-Saharan Africa: a multi-country cross-sectional study. Afr J Reprod Health.

[8] Amjad S, MacDonald I, Chambers T, Osornio-Vargas A, Chandra S, Voaklander D, Ospina MB (2019). Social determinants of health and adverse maternal and birth outcomes in adolescent pregnancies: a systematic review and meta-analysis. Paediatr Perinat Epidemiol.

[9] Westenberg L, van der Klis KA, Chan A, Dekker G, Keane RJ (2002). Aboriginal teenage pregnancies compared with non-Aboriginal in South Australia 1995-1999. Aust N Z J Obstet Gynaecol.

[10] Diabelková J, Rimárová K, Dorko E, Urdzík P, Houžvičková A, Argalášová Ľ (2023). Adolescent pregnancy outcomes and risk factors. Int J Environ Res Public Health.

[11] Moni SA, Nair MK, Devi RS (2013). Pregnancy among unmarried adolescents and young adults. J Obstet Gynaecol India.

[12] Pergialiotis V, Vlachos DE, Gkioka E, Tsotra K, Papantoniou N, Vlachos GD (2015). Teenage pregnancy antenatal and perinatal morbidity: results from a tertiary centre in Greece. J Obstet Gynaecol.

[13] Shaw M, Lawlor DA, Najman JM (2006). Teenage children of teenage mothers: psychological, behavioural and health outcomes from an Australian prospective longitudinal study. Soc Sci Med.

[14] Bateman BT, Simpson LL (2006). Higher rate of stillbirth at the extremes of reproductive age: a large nationwide sample of deliveries in the United States. Am J Obstet Gynecol.

[15] Kurup A, Viegas O, Singh K, Ratnam SS (1989). Pregnancy outcome in unmarried teenage nulligravidae in Singapore. Int J Gynaecol Obstet.

[16] Gökçe B, Ozşahin A, Zencir M (2007). Determinants of adolescent pregnancy in an urban area in Turkey: a population-based case-control study. J Biosoc Sci.

[17] Garwood SK, Gerassi L, Jonson-Reid M, Plax K, Drake B (2015). More than poverty: the effect of child abuse and neglect on teen pregnancy risk. J Adolesc Health.

[18] Imamura M, Tucker J, Hannaford P (2007). Factors associated with teenage pregnancy in the European Union countries: a systematic review. Eur J Public Health.

[19] Kassa GM, Arowojolu AO, Odukogbe AA, Yalew AW (2018). Prevalence and determinants of adolescent pregnancy in Africa: a systematic review and Meta-analysis. Reprod Health.

[20] Apinuntavech S (2017). Life assets in teenage pregnancy. Siriraj Med J.

[21] Were M (2007). Determinants of teenage pregnancies: the case of Busia District in Kenya. Econ Hum Biol.

[22] Whitworth M, Cockerill R, Lamb H (2017). Antenatal management of teenage pregnancy. Obstet Gynaecol Reprod Med.

[23] Panda A, Parida J, Jena S, Pradhan A, Pati S, Kaur H, Acharya SK (2023). Perception, practices, and understanding related to teenage pregnancy among the adolescent girls in India: a scoping review. Reprod Health.

[24] Te Lindert L, van der Deijl M, Elirehema A, van Elteren-Jansen M, Chitanda R, van den Akker T (2021). Perceptions of factors leading to teenage pregnancy in Lindi Region, Tanzania: a grounded theory study. Am J Trop Med Hyg.

[25] Ngonzi J, Bebell LM, Boatin AA (2021). Impact of an educational intervention on WHO surgical safety checklist and pre-operative antibiotic use at a referral hospital in southwestern Uganda. Int J Qual Health Care.

[26] Ayanaw Habitu Y, Yalew A, Azale Bisetegn T (2018). Prevalence and factors associated with teenage pregnancy, northeast Ethiopia, 2017: a cross-sectional study. J Pregnancy.

[27] Gupta N, Kiran U, Bhal K (2008). Teenage pregnancies: obstetric characteristics and outcome. Eur J Obstet Gynecol Reprod Biol.

[28] Zhang T, Wang H, Wang X, Yang Y, Zhang Y, Tang Z, Wang L (2020). The adverse maternal and perinatal outcomes of adolescent pregnancy: a cross sectional study in Hebei, China. BMC Pregnancy Childbirth.

[29] Shawky S, Milaat W (2000). Early teenage marriage and subsequent pregnancy outcome. East Mediterr Health J.

[30] Geltore TE, Anore DL (2021). The impact of antenatal care in maternal and perinatal health. Empowering midwives and obstetric nurses.

[31] Kuhnt J, Vollmer S (2017). Antenatal care services and its implications for vital and health outcomes of children: evidence from 193 surveys in 69 low-income and middle-income countries. BMJ Open.

[32] Tekelab T, Chojenta C, Smith R, Loxton D (2019). The impact of antenatal care on neonatal mortality in sub-Saharan Africa: a systematic review and meta-analysis. PLoS One.

[33] World Health Organization (2016). WHO Recommendations on Antenatal Care for a Positive Pregnancy Experience [Internet]. Geneva: World Health Organization. WHO recommendations on antenatal care for a positive pregnancy experience.

[34] Kongwattanakul K, Thamprayoch R, Kietpeerakool C, Lumbiganon P (2020). Risk of severe adverse maternal and neonatal outcomes in deliveries with repeated and primary cesarean deliveries versus vaginal deliveries: a cross-sectional study. J Pregnancy.

[35] Ochieng Arunda M, Agardh A, Asamoah BO (2020). Cesarean delivery and associated socioeconomic factors and neonatal survival outcome in Kenya and Tanzania: analysis of national survey data. Glob Health Action.

[36] Pires-Menard A, Flatley C, Kumar S (2021). Severe neonatal outcomes associated with emergency cesarean section at term. J Matern Fetal Neonatal Med.

[37] Tefera M, Assefa N, Roba KT, Gedefa L (2021). Adverse neonatal outcome are more common among babies born by cesarean section than naturally born babies at public hospitals in eastern Ethiopia: a comparative prospective follow-up study at eastern Ethiopia. Glob Pediatr Health.

[38] Zhang J, Liu X, Rao L, Ma R, Wu W, Chen C, Lin Y (2023). Adverse obstetric and perinatal outcomes of patients with history of recurrent miscarriage: a retrospective cohort study. Fertil Steril.

[39] Patel K, Pirie D, Heazell AE, Morgan B, Woolner A (2024). Subsequent pregnancy outcomes after second trimester miscarriage or termination for medical/fetal reason: a systematic review and meta-analysis of observational studies. Acta Obstet Gynecol Scand.

[40] Fawzy M, Saravelos S, Li TC, Metwally M (2016). Do women with recurrent miscarriage constitute a high-risk obstetric population?. Hum Fertil (Camb).

[41] Gunnarsdottir J, Stephansson O, Cnattingius S, Akerud H, Wikström AK (2014). Risk of placental dysfunction disorders after prior miscarriages: a population-based study. Am J Obstet Gynecol.

[42] Jauniaux E, Hempstock J, Greenwold N, Burton GJ (2003). Trophoblastic oxidative stress in relation to temporal and regional differences in maternal placental blood flow in normal and abnormal early pregnancies. Am J Pathol.

[43] Plaisier M (2011). Decidualisation and angiogenesis. Best Pract Res Clin Obstet Gynaecol.

[44] Wu CQ, Nichols K, Carwana M, Cormier N, Maratta C (2022). Preterm birth after recurrent pregnancy loss: a systematic review and meta-analysis. Fertil Steril.

[45] Bhattacharya S, Townend J, Bhattacharya S (2010). Recurrent miscarriage: are three miscarriages one too many? Analysis of a Scottish population-based database of 151,021 pregnancies. Eur J Obstet Gynecol Reprod Biol.

[46] Oladapo OT, Diaz V, Bonet M (2018). Cervical dilatation patterns of 'low-risk' women with spontaneous labour and normal perinatal outcomes: a systematic review. BJOG.

[47] Shah PS, Balkhair T, Ohlsson A, Beyene J, Scott F, Frick C (2011). Intention to become pregnant and low birth weight and preterm birth: a systematic review. Matern Child Health J.

[48] Gipson JD, Koenig MA, Hindin MJ (2008). The effects of unintended pregnancy on infant, child, and parental health: a review of the literature. Stud Fam Plann.

[49] Omani-Samani R, Ranjbaran M, Mohammadi M, Esmailzadeh A, Sepidarkish M, Maroufizadeh S, Almasi-Hashiani A (2019). Impact of unintended pregnancy on maternal and neonatal outcomes. J Obstet Gynaecol India.

[50] Yoosefi Lebni J, Solhi M, Ebadi Fard Azar F, Khalajabadi Farahani F, Irandoost SF (2023). Exploring the consequences of early marriage: a conventional content analysis. Inquiry.

[51] World Health Organization (2016). World Health Organization. WHO recommendations on antenatal care for a positive pregnancy experience [Internet]. Geneva: World Health Organization; 2016 [cited 2024 Apr 10]. 152 p. Available from. WHO recommendations on antenatal care for a positive pregnancy experience.

[52] Dibaba Y, Fantahun M, Hindin MJ (2013). The effects of pregnancy intention on the use of antenatal care services: systematic review and meta-analysis. Reprod Health.

[53] Goossens J, Van Den Branden Y, Van der Sluys L, Delbaere I, Van Hecke A, Verhaeghe S, Beeckman D (2016). The prevalence of unplanned pregnancy ending in birth, associated factors, and health outcomes. Hum Reprod.

[54] Carlander A, Hultstrand JN, Reuterwall I, Jonsson M, Tydén T, Kullinger M (2023). Unplanned pregnancy and the association with maternal health and pregnancy outcomes: a Swedish cohort study. PLoS One.

